# Arbuscular mycorrhizal symbiosis in tomato roots with a diverse range of carotene accumulation

**DOI:** 10.1007/s00572-026-01277-0

**Published:** 2026-06-10

**Authors:** Luca Giovannini, José Eduardo Marqués-Gálvez, Fabiano Sillo, Domenico De Paola, Angelo Petrozza, Teresa Mango, Donato Melfi, Jian You Wang, Valentina Fiorilli, Filomena Carriero, Raffaella Balestrini

**Affiliations:** 1 Institute of Sustainable Plant Protection, National Research Council of Italy, Strada delle Cacce 73, Torino, 10135 Italy; 2 Institute of Biosciences and Bioresources, National Research Council of Italy, Via G. Amendola 165/A, Bari, 70126 Italy; 3 Mycology-Mycorrhizae-Plant Biotechnology Group, Department of Plant Biology, CEIR Campus Mare Nostrum (CMN), University of Murcia, Campus de Espinardo, Murcia, 30100 Spain; 4 ALSIA Centro Ricerche Metapontum Agrobios, s.s. Jonica 106, km 448,2, Metaponto, MT 75010 Italy; 5 Agricultural Biotechnology Research Center, Academia Sinica Biotechnology Center in Southern Taiwan, Academia Sinica, Tainan, 71110 Taiwan; 6 Department of Life Sciences and Systems Biology, University of Torino, Viale Mattioli 25, Torino, 10125 Italy

**Keywords:** *Solanum lycopersicum*, Arbuscular mycorrhizal fungi, Carotenoids, Transcriptomics, Mutants

## Abstract

Arbuscular mycorrhizal (AM) symbiosis is regulated by carotenoid-derived molecules, including strigolactones and other apocarotenoids. However, the role of root carotene availability remains poorly understood. Here we evaluated AM performance in tomato using two mutants showing contrasting root carotenoid profiles, i.e. *cyc-b7*, an EMS-derived TILLING mutant carrying a characterized mutation in *Cyc-B*, and *7458-Y*, an uncharacterized mutant line showing high carotenoid accumulation), compared to their related wild type (Red Setter). Not-inoculated and AM fungal inoculated plants were grown under controlled conditions and root colonization parameters were assessed after two months. Root carotenoids were quantified by high-performance liquid chromatography (HPLC), and RNA-seq analysis was performed on root samples to understand the tomato response to the inoculation. Roots of *cyc-b7* accumulated significantly lower total carotenoids than Red Setter, including reduced lutein and β-carotene, whereas *7458-Y* showed increased β-carotene, together with higher arbuscule abundance than both Red Setter and *cyc-b7*. Transcriptome profiling revealed a genotype-dependent response to AMF inoculation, with symbiosis-related genes differentially regulated in the two mutant lines. In *cyc-b7*, AMF inoculation was associated with reduced expression of genes encoding nutrient transporters, as well as of a gene encoding a symbiosis receptor-like kinase (SYMRK), a component of the common symbiosis signaling pathway. By contrast, in *7458-Y*, AMF inoculation was associated with up-regulation of a gene encoding a LysM receptor-like kinase involved in AM establishment, and of a gene, *SlD27*, related to strigolactone biosynthesis. Overall, our results support a link between root carotenoid metabolism and AMF colonization.

**Supplementary Information:**

The online version contains supplementary material available at 10.1007/s00572-026-01277-0.

## Introduction

Arbuscular mycorrhizal (AM) symbiosis is one of the most widespread plant-fungus associations in nature, often functioning as a mutualism, although the outcome of the interaction may vary depending on the host plant, fungal partner and environmental conditions (Lanfranco et al. 2016; Balestrini et al. 2024). It consists of the interaction between the roots of over 70% of terrestrial plant species and soil fungi belonging to the *Glomeromycota* clade (Wijayawardene et al. 2024). AM fungi obtain carbon fixed through plant photosynthesis, while the host plant benefits in exchange from enhanced uptake of mineral nutrients (i.e., phosphorus and nitrogen) and water (Wipf et al. 2019). AM symbiosis has shaped plant evolution until present days and played a key role in plant adaptation to terrestrial environments (Rich et al. 2021). Thanks to the widespread nature of the interaction (Stürmer et al. 2018) and to the capacity of improving plant nutrition and enhancing resilience against many biotic and abiotic factors (Balestrini et al. 2024), AM symbiosis has attracted attention for its potential use in sustainable agriculture (Igiehon and Babalola 2017).

To better implement the use of AM fungi in agriculture, more studies are needed to further understand the molecular processes that drive the outcome of the interaction. AM fungi and plant roots establish an intense molecular dialogue both before and during contact, which governs the establishment and development of AM structures for nutrient exchange, i.e., arbuscules, arbusculate coils and hyphal coils (Oldroyd 2013; Bonfante and Genre 2015; Rodríguez-García and Müller 2026). Among the different actors participating in this crosstalk, the plant hormone strigolactones (SLs) play a prominent role. Besides being a hormone, SLs are essential plant-microbe regulators that derive from carotenoid metabolism (Lanfranco et al. 2018). They are a cleavage product of β-carotene, and therefore, their production is linked to carotenoid metabolism and carotene availability as a precursor (Al-Babili and Bouwmeester 2015; Wang et al. 2024). SLs mediate AM interactions by promoting fungal spore germination, enhancing hyphal ramification (Akiyama et al. 2005, 2010; Besserer et al. 2006), inducing the secretion of fungal effectors that promote AM symbiosis, such as *Rhizophagus irregularis* SIS1 (Tsuzuki et al. 2016) and stimulating the production of MYC factors (Genre et al. 2013), which overall facilitate the interaction. Other carotenoid-derived products (also known as apocarotenoids), such as abscisic acid (ABA), have been implicated in both arbuscule development and the systemic regulation of drought stress responses in arbuscular mycorrhizal plants, and also show systemic changes in abundance (Herrera-Medina et al. 2007; Ren et al. 2019), while cyclohexanone derivatives (i.e., blumenols) and mycorradicins accumulate in arbuscule-containing cells (Strack and Fester 2006; Walter et al. 2010). More recently, in rice, other carotenoid-derived molecules, including zaxinone and β-ionone, have been identified as playing a role in the establishment of AM symbiosis (Wang et al. 2019; Fiorilli et al. 2019; Votta et al. 2024), suggesting a significant role for carotenoid metabolism in AM symbiosis establishment. Consistently, Giudice et al. (2026), focusing on pea, reported that CCD7 might be involved in AM colonization independently of canonical SL biosynthesis, suggesting a possible link with other apocarotenoid pathways, including zaxinone-related processes.

Because beneficial plant-microbe interactions depend on host genetic control of signaling and metabolic processes, TILLING (Targeting Induced Local Lesions in Genomes) lines are particularly useful to dissect how specific loci shape colonization outcomes and symbiosis establishment, as well as to link metabolic traits to symbiosis performance (Perry et al. 2009; Keymer et al. 2017). TILLING mutant collections provide a powerful non-transgenic reverse-genetics resource, generating allelic series of point mutations in a defined crop background and allowing functional tests of candidate genes without introducing transgenes (McCallum et al. 2000). In *Solanum lycopersicum* L., publicly available EMS (ethyl methanesulfonate)-TILLING platforms (including genotypes MicroTom and Red Setter) have expanded the toolbox for linking genotype to phenotype across diverse pathways, from development and hormone signaling to metabolism (Minoia et al. 2010; Okabe et al. 2011).

Here, to decipher the effect of carotene accumulation/depletion on AM symbiosis, we have evaluated the mycorrhizal capacity of two different *S. lycopersicum* EMS mutant lines, i.e. *cyc-b7* and *7458-Y*, which show differential degrees of carotenoid accumulation in roots and fruits. The tomato mutant *cyc-b7* was previously characterized by Silletti et al. (2013) to have a nucleotide substitution (A949G) in the *chromoplast-specific lycopene β-cyclase* (*cyc-b*) gene, which was identified as a missense mutation that caused an amino acid substitution (R317G). This protein is mainly responsible for the conversion of lycopene to β-carotene and plays an important role in fruit ripening (Pecker et al. 1996). This substitution was predicted to affect the activity of the protein encoded by *cyc-b* and, in fact, the authors showed that the presence of this allele increased total lycopene content in fruits without altering β-carotene levels significantly (Silletti et al. 2013). Results, however, indicate that this effect may be dependent of tissue, since, in roots, this mutation caused decreased concentrations of β-carotene. Moreover, the protein encoded by *cyc-b* has been reported to be a putatively bifunctional carotenoid enzyme, capable of catalyzing both lycopene cyclization to β-carotene and violaxanthin conversion to neoxanthin (Hirschberg 2001). However, subsequent functional characterization studies have not provided evidence supporting this hypothesis. As reported by Ronen et al. (2000) and Stigliani et al. (2011), *cyc-b* encodes a lycopene β-cyclase that functions in chromoplast-containing tissues and does not participate in the violaxanthin-to-neoxanthin conversion in green tissues. On the other hand, *7458-Y* mutant line is an uncharacterized mutant line showing high carotenoid accumulation in fruits and roots. Our study suggests that, with moderate effects, root carotenoid accumulation significantly enhances arbuscule formation, whereas lower concentrations are associated with decreased colonization. Finally, we also performed transcriptomic analyses of both not-inoculated and AMF-inoculated roots to gain insight into the potential transcriptional mechanisms underlying differential carotene accumulation and the associated mycorrhizal phenotypes.

## Materials and methods

### Biological material and root carotenoid accumulation analysis

Three different tomato (*S. lycopersicum*) genotypes were used in this study: two EMS mutants, *cyc-b7 and 7458-Y* lines, and the Red Setter cultivar, used as the control. Particularly, *cyc-b7* line was selected by TILLING molecular screenings, which were carried out on the tomato Red Setter cultivar-based TILLING platform (Minoia et al. 2010) and it was previously characterized to carry the missense mutation A949G in *CYC-B* allele (Silletti et al. 2013). Conversely, *7458-Y* line was visually selected in an open field trial for its mutant phenotype, i.e., its orange fruits (Supplementary Fig. S1). The genetic lesion(s) underlying the *7458-Y* fruit color mutant phenotype remains unknown, and the causal mutation(s) have not yet been identified. Seeds of the two mutant lines *cyc-b7* and *7458-Y* belong to the tomato mutant collection developed and available at Alsia-Metapontum Agrobios Research Center (http://www.alsia.it/tilling). For carotenoid analysis, plants (only not inoculated) were grown in pots under greenhouse conditions under a 12 h light/12 h dark photoperiod, with day/night temperatures of 22/18°C. Environmental conditions were maintained within the standard range generally recommended for tomato growth (approximately 60–70% RH). Plants received a nutrient solution containing Master 13.40.13 fertilizer (Valagro SpA) at 1 g L⁻¹. Roots were collected at the fruiting stage and analyzed for carotenoid content as described below. The *7458-Y* line and the corresponding Red Setter control were grown from March to June 2021, whereas *cyc-b7* and the corresponding Red Setter control were grown from July to October 2023. It should be noted that the two experiments were conducted in different periods because the two mutant lines were identified and phenotyped at different times. For this reason, it was not possible to carry out the experiments under the same supplemental lighting conditions: in 2021, plants were maintained under 600 W sodium lamps (OSRAM), whereas in 2023 plants were grown under 90 W E40 6400 K LED lamps (DURALAMP). Since the two mutant lines were grown in different experimental periods, each line was evaluated relative to its corresponding Red Setter control cultivated under the same conditions and sampled at the same developmental stage. Accordingly, carotenoid data were interpreted within each experimental set. The carotenoid contents of roots of experimental lines were determined by high-performance liquid chromatography (HPLC) analysis at the fruiting phenological stage. The roots were freeze-dried and ground using a ball mill. The resulting material was then extracted with 20 mL of a mixture of hexane: acetone: ethanol (50:25:25). The mixture was stirred for approximately 4 h and centrifuged for 10 min at 4000 rpm; the pellet was discarded and 2.5 mL of 8% (W/V) KOH was added to the supernatant. After 5 min, 2.5 mL of NaCl (25%) was added. The saponified material was extracted by adding 10 mL of hexane and centrifuged for 10 min at 4 °C at 4000 rpm. The upper phase was retained, and additional 10 mL of hexane was added to the lower phase to repeat the extraction. The two extracts were combined and evaporated under nitrogen. The pellet was resuspended in 1 mL of ethyl acetate. Pigment separation was performed with an Agilent 1200 Chemstation HPLC system equipped with a DAD system and a C30 reverse-phase column (C30-YMC, 250 mm 9 4.6 mm, S-5 lm). Data acquisition and analysis were carried out with the Chemstation for LC 3-D system software. Carotenoids were identified by their characteristic absorption spectra and typical retention times. Quantification of carotenoid compounds was performed using calibration curves generated from authentic standards. Lutein, β-carotene and 80-apo- β-carotenal (internal standard) were purchased from Sigma–Aldrich. Each determination was performed in triplicate on three biological replicates for each tissue sample.

### Experimental setup for the mycorrhizal inoculation and colonization assessment

Seeds of the three tomato genotypes were germinated in trays for 19 days before starting the experiment. Seeds were germinated in a sandy medium consisting of 70% sand and 30% peat. Then, 20 seedlings from each genotype were transferred to cone-shaped pots (12 cm in height, with an upper diameter of 14 cm and a lower diameter of 12 cm, corresponding to a volume of approximately 1370 cm³) with 1 kg of substrate, and half of them were also inoculated (called MYC) with a layer of mycorrhizal inoculum (*Funneliformis mosseae* (T.H. Nicolson & Gerd.) C. Walker & A. Schüßler, MycAgro Lab, Bretenière, France; it contained a minimum of 10 active propagules/g) diluted 1:3 with the substrate. The experimental substrate consisted of a sandy soil mixture composed of 70% sand and 30% peat (Vigorplant S.r.l., Italy). Prior to use, the substrate was sterilized by autoclaving at 121 °C for 20 min. Overall, six different treatments with 10 plant replicates each were set up: Red Setter not-inoculated (hereinafter referred as NMYC), Red Setter inoculated with AMF (hereinafter referred as MYC), *cyc-b7*_NMYC, *cyc-b7*_MYC, *7458-Y*_NMYC, *7458-Y*_MYC. The pots were arranged on the greenhouse bench following a completely randomized design. Plants were irrigated with water for one month and then with ½-strength Hoagland’s solution supplemented with KH₂PO₄ (1 mL L⁻¹ of a 0.1 M stock solution) for another month. Two months after seedling inoculation, mycorrhizal colonization was evaluated on ten plants *per* genotype. The roots were carefully rinsed with tap water and treated with 10% KOH for 10 min at 80 °C. After clearing, roots were thoroughly washed under running tap water to remove residual KOH and then stained overnight at room temperature with 0.1% cotton blue in lactic acid. After staining, the roots were rinsed three times with tap water and preserved in lactic acid. For each biological replicate (*n* = 10), twenty 1-cm-long root fragments were mounted on each of three microscope slides (20 fragments *per* slide, for a total of 60 fragments *per* replicate). Mycorrhizal colonization was assessed according to Trouvelot et al. (1986). The frequency of mycorrhization (F%), the intensity of mycorrhization (M%), and the arbuscule abundance in mycorrhizal root parts (a%), and in the whole root system (A%) were determined based on microscopic observation of fungal structures (hyphae, arbuscules, and vesicles) under a light microscope.

### RNA extraction and sequencing

Two months after seedling inoculation, 100 mg of root material from each of three individual biological replicates *per* genotype and treatment were collected and immediately frozen in liquid nitrogen. These samples were stored at -80 °C until RNA extraction. Total RNA was extracted according to the cetyltrimethylammonium bromide-based method of Chang et al. (1993). Quantity and quality of the extracted RNA samples were determined using a Nanodrop 2000 spectrophotometer (Thermo Fisher Scientific, Waltham, MA, USA). Library preparation and RNA sequencing were performed by IGATech s.r.l Laboratories (Udine, Italy). In detail, a Universal Plus mRNA-Seq kit (Tecan Genomics, Redwood City, CA, USA) was used for library preparation following the manufacturer’s instructions and sequencing was performed on Illumina NovaSeq 6000 apparatus (Illumina, San Diego, CA, USA). Trimming of lower quality bases and adapters was performed using ERNE software (Del Fabbro et al. 2013). Raw reads were processed and mapped against *S. lycopersicum* genome (https://solgenomics.net, SL4.0) using SAMtools v1.22.1 (Danecek et al. 2021) and hisat2 v2.2.1 (Kim et al. 2019), raw gene counts were calculated using featureCounts v.2.1.1 (Liao et al. 2014) and they were normalized, visualized and analyzed using DESeq2 (Love et al. 2014). For a gene to be considered differentially expressed, we considered those with a log2 fold change ≥ 1 and a false discovery rate (FDR) ≤ 0.05. Gene Ontology enrichment was performed using TOPGO v2.62.0 package (Alexa and Rahnenführer 2025) and gene overlaps were performed using UpsetR v1.4.0 package (Conway et al. 2017). To assess the presence of fungal RNA in AMF-inoculated roots, reads not aligned to the *S. lycopersicum* genome were retrieved from both MYC and NMYC samples and mapped against the *F. mosseae* reference genome available from ENA under BioProject accession PRJEB45340 (Montoliu-Nerin et al. 2021). Mapping and quantification were performed using Salmon v.1.4.0 (Patro et al. 2017), and Salmon outputs were imported into R using tximport v.1.22.0 (Soneson et al. 2016). The number of reads assigned to *F. mosseae* transcripts was calculated for each sample and expressed relative to the reads unmapped to the tomato genome. NMYC samples were used as controls to estimate background mapping to the fungal reference. Recurrently detected *F. mosseae* transcripts were inspected together with their functional annotations to support the presence of fungal transcripts in MYC samples.

### Mutation analysis

Variant calling was performed on processed and mapped reads using FreeBayes v1.3.10 (Garrison and Marth 2012), using the reference genome as input and assuming diploidy. Variant detection was restricted to sites supported by a minimum of three alternate reads, with a minimum alternate allele fraction of 0.08. Only reads with a minimum mapping quality of 20 and bases with a minimum base quality of 20 were considered. VCF files were indexed with bcftools v1.22 (Danecek et al. 2021). Allele frequency (AF) annotations were added, and variants were filtered excluding sites with a quality score below 30, a read depth (DP) below 10, or an alternate allele frequency below 0.1. Mutation type was manually assessed with the help of Integrative Genomics Viewer (IGV) (Robinson et al. 2017) and Translate tool from Expasy (https://web.expasy.org/translate/).

### Statistical analysis

Carotenoid accumulation data were analyzed using Welch’s t-test to evaluate differences between means of each mutant vs. the Red Setter control without assuming equal variances. Since mycorrhizal colonization parameters are percentage data, values were arcsine square-root transformed before statistical analysis, to meet the assumptions of normality of residuals and homogeneity of variances (verified using Shapiro-Wilk and Levene tests, respectively). Then, mycorrhizal colonization data were analysed using pairwise one-way ANOVA followed by Tukey HSD. Statistical significance was determined at *p* < 0.05. Statistical analyses were performed in R v4.4.0 (R Core Team, 2016).

## Results

### Red Setter, *cyc-b7* and *7458-Y Solanum lycopersicum* genotypes differentially accumulate β-carotene in roots

Carotenoid content was quantified by HPLC in *S. lycopersicum* roots of wild type (Red Setter) and mutant (*cyc-b7* and *7458-Y*) genotypes (Supplementary Table S1). As described in the Materials and Methods, the two mutant lines were analysed in separate experimental sets. Therefore, carotenoid accumulation in each mutant was interpreted relative to the Red Setter plants grown in parallel under the same conditions, rather than by directly comparing absolute carotenoid levels between the two mutant experiments. In particular, the mutant *cyc-b7* showed a significant decrease in total carotenoids (log2FC = -0.84) compared to Red Setter roots from plants grown in parallel, including lutein (log2FC = -1.42), β-carotene (log2FC = -0.98), and other unidentified carotenoids (log2FC = -0.65) (Table 1 and Supplementary Table S1a). On the other hand, in a separate experiment, *7458-Y* showed higher total carotenoid content (log2FC = 1.73) than Red Setter. While in this case, lutein content was not significantly increased, both β-carotene (log2FC = 1.82) and other unidentified carotenoids (log2FC = 1.62) were significantly more abundant in the *7458-Y* genotype than in Red Setter (Table 1 and Supplementary Table S1b). *7458-Y* genotype has not been previously characterized, and therefore, the genetic basis of the observed carotenoid phenotype remains elusive. Using variant-calling software, we attempted to identify genetic variants in our RNA-seq dataset. When overlapping our variant calling results, we found a total of 13 putative variants exclusively in *7458-Y* genotype (Table 2). Out of the 13 putative variants, 10 were single-nucleotide substitutions, two single base deletions, and one single base insertion. Most of the mutations (nine) resulted in either 5’UTR and 3’UTR region variants, whereas the other four mutations caused missense mutations (Table 2).

**Table 1:** Root carotenoid classes in *Solanum lycopersicum* mutants, expressed as log2 fold change (log2FC) relative to their corresponding Red Setter controls. Data were obtained from two independent experiments, one for each mutant line (*cyc-b7* and *7458-Y*), with a separate Red Setter control in each experiment. The log2FC values were calculated from the mean values for each carotenoid class

	Lutein	β-Carotene	Other carotenoids	Total carotenoids
log2FC (*cyc-b7* vs. Red Setter)	-1.42 ± 0.21	-0.98 ± 0.12	-0.65 ± 0.11	-0.84 ± 0.11
log2FC (*7458-Y* vs. Red Setter)	2.34 ± 1.11	1.82 ± 0.35	1.62 ± 0.37	1.73 ± 0.34

**Table 2:** Allelic variants exclusively identified in *7458-Y Solanum lycopersicum* genotype

Genomic coordinate (Chromosome position)	Gene ID	Annotation	Reference allele	Alternative allele	Allele Frequency	Mutation type
SL4.0ch01_71375814	*Solyc01g079690*	ATP-dependent DNA helicase Snf22	T	C	1	Substitution; Missense mutation Tyrà Cys
SL4.0ch04_33236277	*Solyc04g045565*	Unknown protein	CAAAAGGGC	CAAAGGGC	1	Deletion; 5’UTR variant
SL4.0ch04_58775702	*Solyc04g074890*	Unknown protein	C	T	1	Substitution; 5’UTR variant
SL4.0ch04_61721612	*Solyc04g079180*	Unknown protein	C	T	1	Substitution; Missense mutation Arg à Ile
SL4.0ch05_2426977	*Solyc05g007960*	Protein of unknown function DUF246	GAAAAAAAAAATGAAAGAA	GAAAAAAAAATGAAAGAA	0.5	Deletion; 3’UTR variant
SL4.0ch06_41499012	*Solyc06g071250*	Unknown protein	CAAAAAAAAACCACCT	CAAAAAAAAAACCACCT	1	Insertion; 5’UTR variant
SL4.0ch07_2366533	*Solyc07g007755*	Defensin	C	T	0.5	Substitution; 3’UTR variant
SL4.0ch08_27443357	*Solyc08g023460*	Cell cycle control protein	C	T	1	Substitution; 3’UTR variant
SL4.0ch10_38867340	*Solyc10g047130*	RNA-binding protein-like RZ1AL	G	A	1	Substitution; 3’UTR variant
SL4.0ch11_778740	*Solyc11g005910*	Phosphatidylinositol kinase	C	T	0.5	Substitution; Silent mutation
SL4.0ch11_10507138	*Solyc11g020190*	Unknown protein	T	C	1	Substitution; 3’UTR variant
SL4.0ch11_24095495	*Solyc11g032100*	MADS box transcription factor	A	G	1	Substitution; 5’UTR variant
SL4.0ch12_7547198	*Solyc12g017830*	Section 7 guanine nucleotide exchange factor	C	T	1	Substitution; Missense mutation Leu à Phe

### Differential β-carotene accumulation in roots influences AM colonization

To assess the impact of altered carotenoid accumulation on AMF colonization, Red Setter, *cyc-b7*, and *7458-Y* plants were inoculated with *F. mosseae* and analyzed after two months from inoculation.

**Fig. 1:**
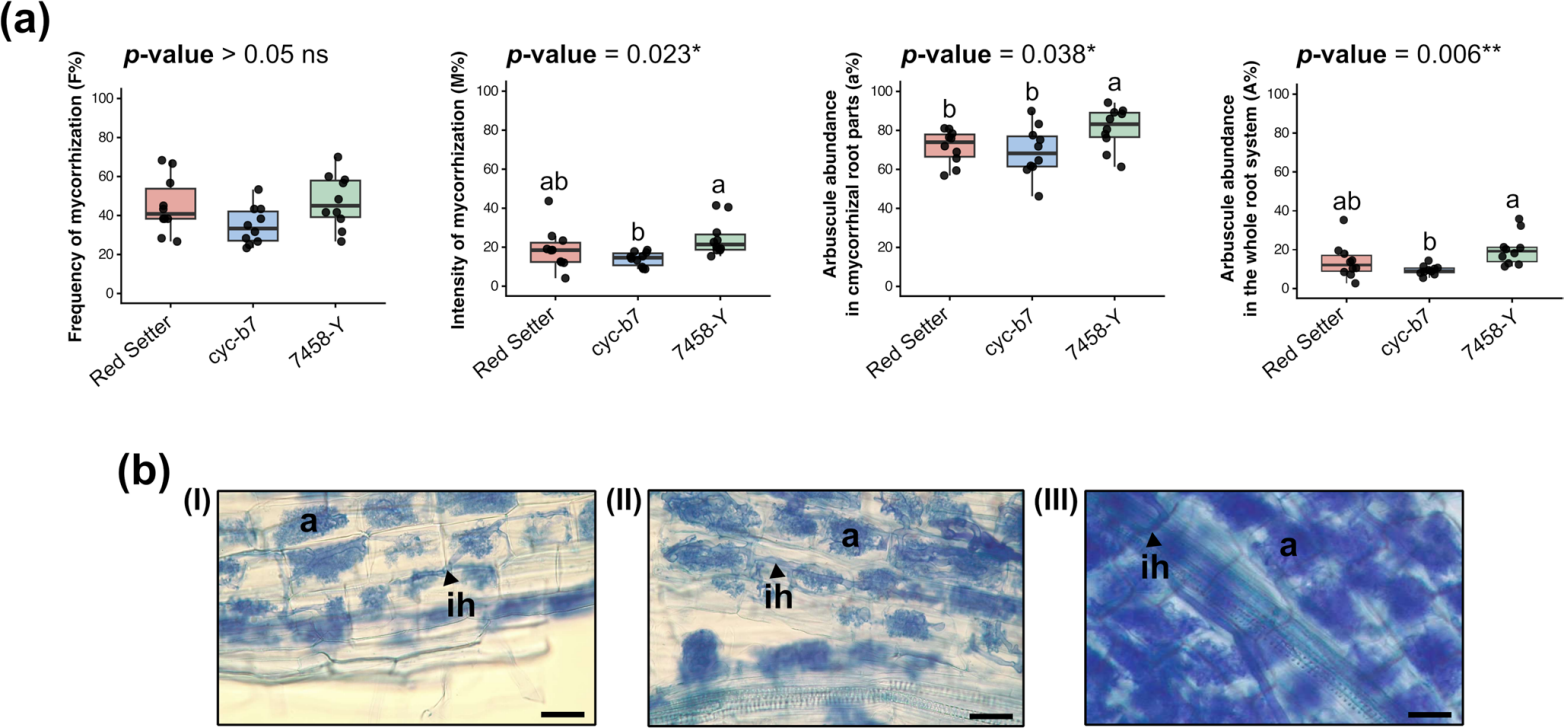
Arbuscular mycorrhizal colonization in different *Solanum lycopersicum* genotypes. **a** Boxplots showing frequency of mycorrhization (F%), intensity of mycorrhization (M%), arbuscule abundance in mycorrhizal root parts (a%), and in the whole root system (A%) of Red Setter, *cyc-b7*, and *7458-Y* plants. Whiskers indicate 1.5× the interquartile range. Individual data points are shown. Asterisks indicate statistically significant differences according to one-way ANOVA followed by Tukey’s HSD test (*p ≤ 0.05, **p ≤ 0.01, ***p ≤ 0.001; ns, not significant). *n* = 10. **b** Representative images of AMF-inoculated roots showing arbuscule formation in (I) Red Setter, (II) *cyc-b7*, and (III) *7458-Y*. a, arbuscules; ih, intraradical hyphae. Scale bars: 30 μm (I, II) and 15 μm (III)

One-way ANOVA showed no significant effect of genotype on mycorrhizal frequency (F%; *p* > 0.05). By contrast, genotype significantly affected mycorrhizal intensity (M%; *p* = 0.0230) and arbuscule abundance in mycorrhizal root parts (a%; *p* = 0.0377), and in the whole root system (A%; *p* = 0.0060). Quantitative assessment revealed that the *cyc-b7* mutant, characterized by reduced root carotenoid levels, showed significantly lower mycorrhization intensity than the *7458-Y* genotype, which displays higher root carotenoid content (Fig. 1a and Supplementary Table S2). Particularly, *cyc-b7* showed a 1.78-fold decrease in mycorrhizal intensity in comparison to *7458-Y*. In addition, Tukey HSD indicated that arbuscule abundance was significantly higher in *7458-Y* compared with both the Red Setter (1.13-fold increase) and *cyc-b7* (1.17-fold increase) (Fig. 1a). Similarly, A% was significantly higher in *7458-Y* than in *cyc-b7* (2.14-fold higher), with Red Setter showing an intermediate phenotype. Representative microscopic observations were consistent with the higher arbuscule abundance in mycorrhizal root parts (a%) observed in *7458-Y*, showing more abundant arbuscular structures than in Red Setter and *cyc-b7* mutant plants (Fig. 1b and Supplementary Table S2).

### Transcriptomic profiles of wild type and mutants in the absence and presence of AMF

**Fig. 2:**
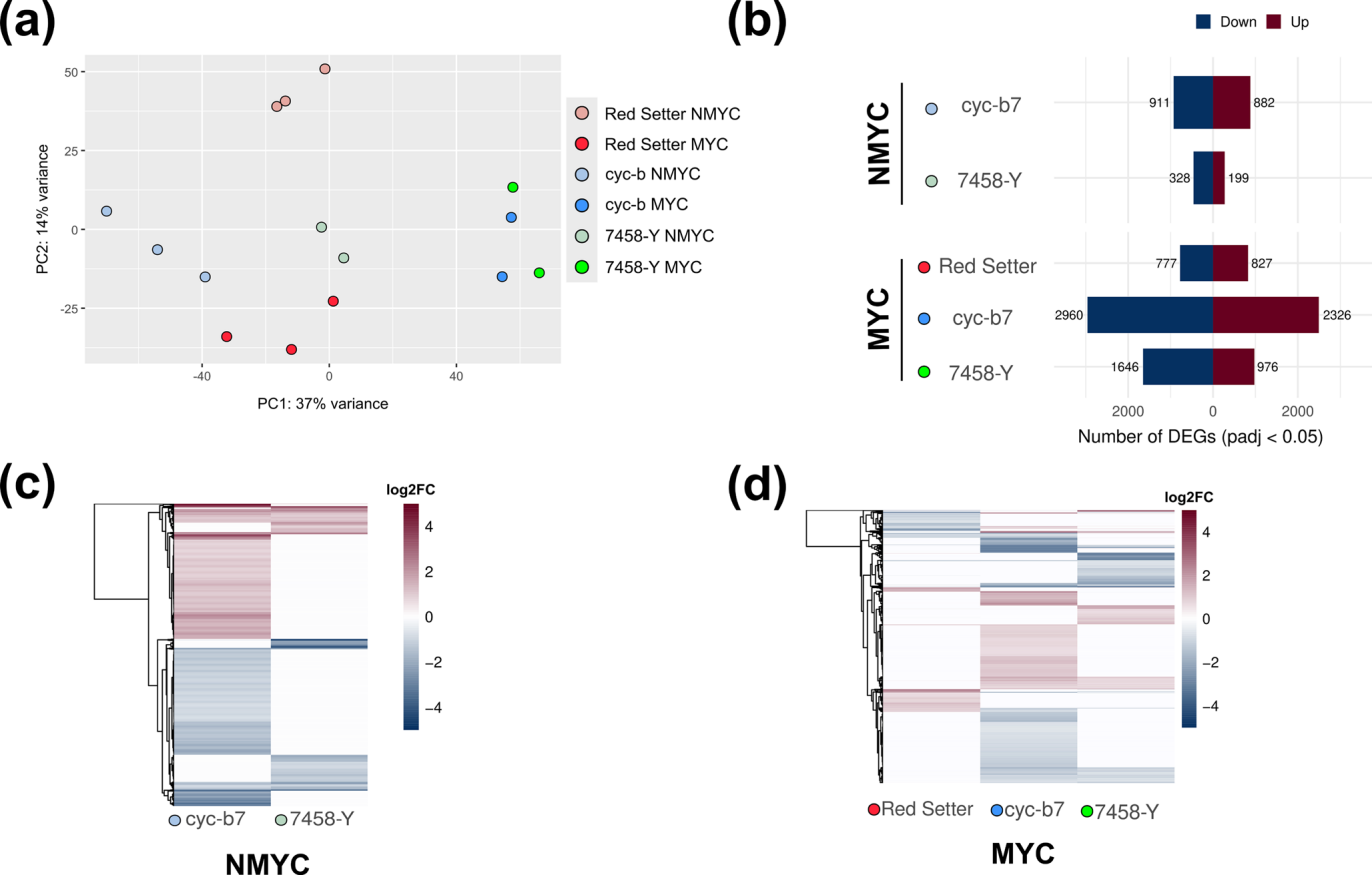
Transcriptomic profiles of the assessed tomato genotypes, not inoculated and inoculated with AMF. **a** Principal component analysis (PCA) of normalized read count from Red Setter and the mutants *cyc-b7* and *7458-Y*. **b** Number of differentially expressed genes (DEGs; *p*-adjusted value < 0.05) showing down (blue) and up regulation (red) in the main comparisons (not-inoculated mutants vs. not-inoculated Red Setter; AMF inoculated genotypes vs. their associated not inoculated controls). **c**, **d** Heatmaps of DEGs (log2FC) with hierarchical clustering, summarizing genotype-dependent transcriptional changes in not-inoculated plants (**c**) and AMF inoculated plants (MYC) (**d**)

The RNA-seq of root samples produced an average of 38.2 million raw reads per sample (range 25.8–50.8 million). Mapping steps on ITAG 4.0 *S. lycopersicum* genome showed an average read mapping rate of 75.87% (range 64.03–83.75%) (Supplementary Table S3). Normalized read counts are reported in Supplementary Table S4. The PCA of normalized read counts (Fig. 2a) showed that PC1 (37% of the total variance) separated the MYC samples of the two mutants (*cyc-b7* MYC and *7458-Y* MYC), which clustered together on the positive side of PC1, from their corresponding not-inoculated controls and from the Red Setter samples. PC2 (14% of the total variance) mainly distinguished Red Setter MYC from Red Setter not-inoculated plants, suggesting a genotype-specific MYC effect in the wild type. Overall, based on normalized read counts, despite their opposite carotenoid phenotypes, *cyc-b7* and *7458-Y* displayed a convergent transcriptomic profile under MYC (Fig. 2a). However, in the PCA, PC1 and PC2 explained only 51% of the total variance, and a substantial fraction of variability (around 49%) remained distributed across additional components. For this reason, PCA was used as an exploratory visualization and interpreted together with the differential expression analyses.

To disentangle genotype-driven transcriptional differences independently of AMF inoculation, comparison of transcriptomic profiles of not-inoculated plants of *cyc-b7* and *7458-Y* with the Red Setter showed that the line *cyc-b7* regulated more genes compared to the wild type than the *7458-Y* genotype, with almost 1,800 genes (911 down-regulated and 882 up-regulated genes, respectively; Fig. 2b-c and Supplementary Tables S5-S6). When comparing RNA-seq data in mycorrhizal roots vs. not-inoculated roots of each genotype, 827 up and 777 down-regulated genes in Red Setter, 2,326 up and 2,960 down-regulated genes in *cyc-b7*, and 976 up and 1,646 down-regulated genes in *7458-Y* genotype, were identified (Fig. 2b-d and Supplementary Tables S7-S9). Gene ontology enrichment of each dataset did not include any term related to carotenoid metabolism or mycorrhizal symbiosis (Supplementary Tables S10-S14).

In the baseline comparisons between not-inoculated mutants and Red Setter, both mutants showed genotype-specific transcriptional differences. In *cyc-b7* not-inoculated vs. Red Setter not-inoculated, the most evident changes involved phytohormone-related genes, secondary metabolism, and oxidative enzymes (Supplementary Table S5). Among the most up-regulated genes, *cyc-b7* showed up-regulation of genes encoding the ethylene biosynthetic enzyme SlACC oxidase 5 (*Solyc07g026650*, log2FC = 4.98), an auxin efflux carrier (*Solyc02g082450*, log2FC = 3.50), and the F-box protein MAX2 (*Solyc12g010900*, log2FC = 1.25), involved in SL signaling/perception (Nelson et al. 2011).

In *7458-Y* not-inoculated vs. Red Setter not-inoculated, the transcriptional profile was mainly characterized by the up-regulation of genes associated with carbon transport/osmolyte metabolism, redox/oxidative metabolism, and hormone-associated responses (Supplementary Table S6). Among the most up-regulated genes, *7458-Y* showed up-regulation of a gene encoding sugar transporter protein 10 (*Solyc03g006650*, log2FC = 5.74), together with genes associated with osmolyte/redox metabolism and ethylene-, auxin-, and ABA-related responses.

Despite these differences, some similarities were observed across the mutants, as both comparisons included DEGs among genes encoding transport-related functions and transcriptional regulators, including the up-regulation of *Solyc09g075040*, which encodes an SPX domain-containing protein involved in phosphate (Pi) homeostasis (Secco et al. 2012; Wang et al. 2021) (log2FC = 2.45 and 2.82 in *cyc-b7* and *7458-Y*, respectively). Particularly, *cyc-b7* showed up-regulation of genes encoding sugar/solute transporters and down-regulation of several genes encoding NPF/NRT1-PTR nitrate transporters. In *7458-Y*, the DEG set included both up-regulated genes encoding sugar/solute transporters and down-regulated genes encoding transport/signaling components, including a SWEET bidirectional sugar transporter (*Solyc03g007360*, log2FC = -4.80), with the complete gene lists reported in Supplementary Tables S5-S6.

In the comparison between AMF-inoculated plants and their corresponding not-inoculated controls, the transcriptional response of AM-responsive transporter genes was strongly genotype-dependent. Several genes encoding transporters previously identified as AM-responsive in tomato roots (Balestrini et al. 2019) were identified as DEGs, with 14 DEGs in Red Setter, 24 in *cyc-b7*, and 13 in *7458-Y* (Supplementary Table S15).

In Red Setter MYC vs. Red Setter not-inoculated, up-regulated genes mainly encoded mineral nutrient and water/solute transporters, including nitrate, sugar, potassium, sulphate, ammonium and tonoplast intrinsic protein transporters, whereas down-regulated genes encoded membrane transport components, including major facilitator superfamily proteins, phosphate and sugar transporters, amino acid transporters, aquaporins, potassium transporters, and tonoplast intrinsic proteins.

In *cyc-b7* MYC vs. *cyc-b7* not-inoculated, only two genes encoding nutrient transporters in this list were up-regulated, whereas 22 were down-regulated, including genes encoding NPF/PTR family members, sugar, phosphate, and proline transporters.

In *7458-Y* MYC vs. *7458-Y* not-inoculated, the only up-regulated gene in this transporter set encoded a SWEET bidirectional sugar transporter (*Solyc03g007360*, log2FC = 2.69), whereas 12 genes were down-regulated, including genes encoding NPF2.9, phosphate, potassium, and lipid A export transporters. The complete gene list is reported in Supplementary Table S15.

Focusing on a set of symbiosis-related genes reported in tomato roots by Balestrini et al. (2019) and Zeng et al. (2023), differential expression under MYC vs. not-inoculated conditions showed a genotype-dependent pattern (Supplementary Table S16). In Red Setter MYC, up-regulation was observed for selected genes encoding a transport- and lipid-related, including a PHO1-like phosphate transporter (*Solyc02g088220*, log2FC = 1.66). In *cyc-b7* MYC, AMF inoculation was associated with down-regulation of genes encoding a purple acid phosphatase (*Solyc01g068380*, log2FC = -2.21), SlSUT2 (*Solyc05g007190*, log2FC = -1.29), and SlSYMRK, a component of the common symbiosis signaling pathway (SYMRK) (*Solyc02g091590*, log2FC = -1.67). In the same genotype, up-regulated genes mainly included lipid metabolism/transport- and signaling-related genes, such as a gene encoding a 3-oxoacyl-[acyl-carrier-protein] synthase (*Solyc08g082620*, log2FC = 1.88). By contrast, in *7458-Y* MYC, AMF inoculation was associated with up-regulation of a gene encoding a NOPE1-like (*Solyc03g080020*, log2FC = 2.95), together with genes encoding the LysM receptor-like kinase SlLYK12 (*Solyc02g081050*, log2FC = 2.10) and an additional LysM domain receptor-like kinase (*Solyc03g121050*, log2FC = 1.33). Additional up-regulated genes in *7458-Y* included genes associated with stress/defense and cell wall-related functions, including a gene encoding a chitinase (*Solyc02g082960*, log2FC = 2.30) (Supplementary Table S16).

**Fig. 3:**
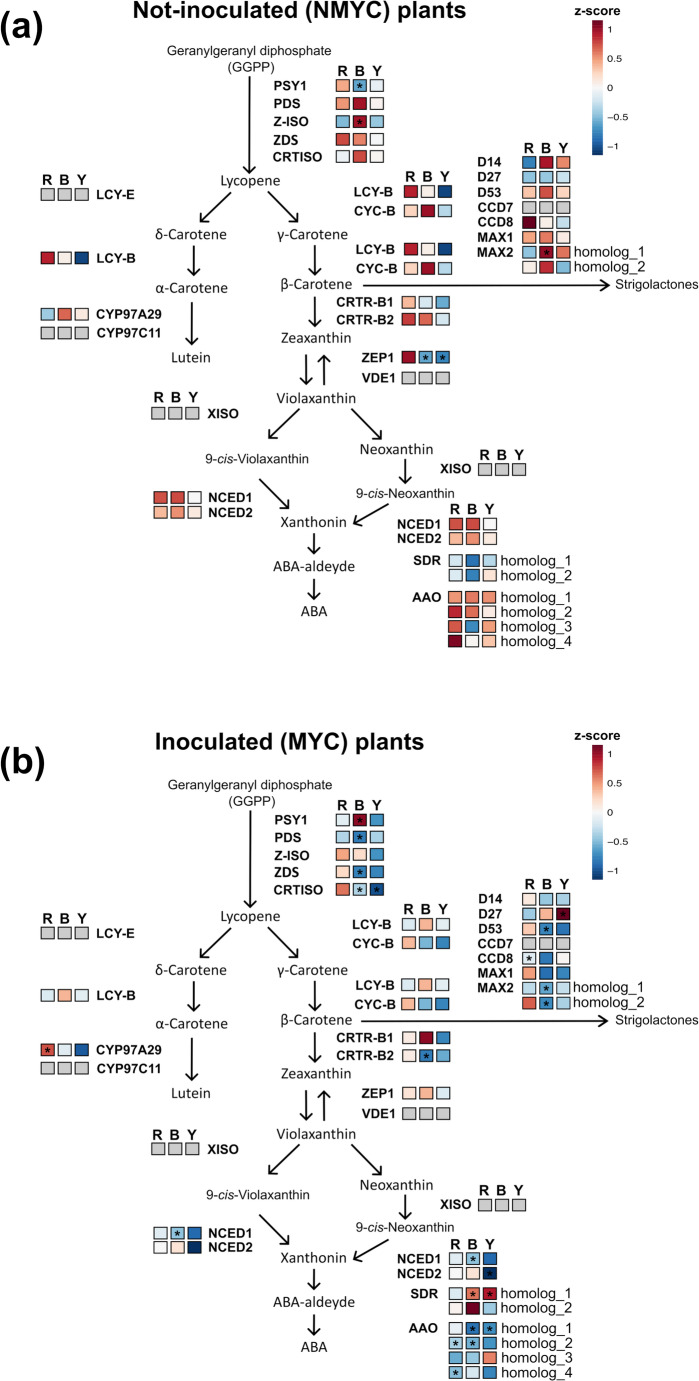
Carotenoid pathway transcription in tomato roots. Schematic overview of carotenoid biosynthesis from geranylgeranyl diphosphate (GGPP) and downstream branches leading to ABA and strigolactone biosynthesis in (**a**) not-inoculated (NMYC) and (**b**) inoculated (MYC) plants. For each gene, the three adjacent squares report row-scaled transcript abundance (normalized read count) in Red Setter (R), *cyc-b7* (B), and *7458-Y* (Y). Where present, gene homologs are labeled as *homolog_n.* Asterisks (*) within individual squares indicate genes identified as differentially expressed genes (DEGs) in the specific comparison represented by that square: in NMYC plants, B_NMYC vs. R_NMYC or Y_NMYC vs. R_NMYC; in MYC plants, R_MYC vs. R_NMYC, B_MYC vs. B_NMYC, or Y_MYC vs. Y_NMYC. Further details are reported in Supplementary Table S17. Dashed arrows indicate putative isomerization steps

To provide an overview of transcriptional patterns across carotene biosynthesis and downstream apocarotenoid branches, we examined normalized read counts for 26 genes involved in carotenoid metabolism (Fig. 3). Nineteen of these genes were significantly differentially expressed in at least one of the conditions tested, whereas the remaining seven genes were not differentially expressed in any comparison (Supplementary Table S17). Among SL-pathway genes, two tomato homologs encoding the F-box protein MAX2 (*Solyc12g010900*, *Solyc07g055120*), were identified as expressed. In not-inoculated tomato mutants, both *SlMAX2* homologs showed the highest relative abundance in *cyc-b7* compared to Red Setter and *7458-Y*, with *Solyc12g010900* detected as a DEG in *cyc-b7* vs. Red Setter (log2FC = 1.25). *SlCCD8* showed a genotype-dependent pattern. In not-inoculated mutants, *SlCCD8* showed the highest relative transcript abundance in Red Setter roots, while it was reduced in *cyc-b7* and was lowest in *7458-Y*. It showed the highest relative transcript abundance in Red Setter roots, while it was reduced in *cyc-b7* and was lowest in *7458-Y*. In AMF-inoculated plants, *SlMAX2* transcripts showed the lowest relative abundance in *cyc-b7* compared with *7458-Y* and Red Setter. Both *SlMAX2* homologs, *Solyc12g010900* and *Solyc07g055120*, were identified as DEGs in *cyc-b7* MYC vs. *cyc-b7* not-inoculated (log2FC = -1.27 and − 1.13, respectively). Transcript abundance of *SlCCD8* remained overall low, resulting in a down-regulated DEG for Red Setter MYC when compared to Red Setter not-inoculated (Supplementary Table S7). In AMF-inoculated plants, the gene encoding DWARF27 (D27), an enzyme that catalyses the isomerization of all-*trans*- into 9-*cis*-β-carotene, thereby initiating SL biosynthesis (Yang et al. 2023), showed high transcript abundance in *7458-Y*. The upstream steps in carotenoid biosynthesis also showed genotype- and AMF-inoculation-dependent effects. In not-inoculated roots, *cyc-b7* showed high transcript abundance of genes coding for enzymes involved in the conversion from geranylgeranyl diphosphate (GGPP) to lycopene, i.e., *SlPDS*, *SlZ-ISO*, *SlCRTISO*, and for *SlCYC-B*, whereas *7458-Y* generally showed low transcript abundance. In MYC plants, transcript abundance of the gene *SlPSY1* was relatively high in *cyc-b7*. The ABA branch showed an overall decrease in transcripts related to *SlNCED1* in MYC roots, particularly in the two mutants, and a general reduction in multiple transcripts associated with *SlAAO* homologs under MYC, again most evident in the mutants. In contrast, *SlSDR* homologs showed genotype-specific modulation under MYC (one homolog increased in *7458-Y* and another in *cyc-b7*; Fig. 3). Differential expression analysis further revealed that, in not-inoculated *cyc-b7* compared with Red Setter, *SlCRTISO* and *SlZ-ISO* were significantly up-regulated, whereas *SlZEP1* was down-regulated. *SlZEP1* was also found to be down-regulated in the *7458-Y* mutant compared to the wild-type Red Setter. Considering mycorrhizal roots, most of the changes accumulated in *cyc-b7* genotype inoculated with AMF (12 DEGs). When comparing mycorrhizal vs. not-inoculated tissue, *cyc-b7* genotype up-regulated *SlPSY1* but down-regulated other genes of carotene biosynthesis, such as *SlPDS*,* SlZDS*, and *SlCRTISO* (Supplementary Table S17). *7458-Y* genotype strongly down-regulated not only *SlCRTISO* but also *SlNCED2*, a key gene in ABA biosynthesis, when comparing MYC vs. NMYC roots. The gene *SlD27* was up-regulated in *7458-Y* when compared to its not-inoculated control. In Red Setter, MYC roots vs. NMYC roots, *SlCCD8* was down-regulated.

To further compare the transcriptional response to AMF inoculation across the three genotypes, we analysed the overlap among DEGs identified in each MYC vs. not-inoculated comparison (Supplementary Fig. S2 and Supplementary Table S18). Only a limited core of DEGs was commonly regulated across Red Setter, *cyc-b7*, and *7458-Y*, including four commonly up-regulated genes and 43 commonly down-regulated genes. Overall, the differences observed among MYC vs. not-inoculated comparisons indicate that AMF inoculation triggered a genotype-specific transcriptional response (Supplementary Fig. S2).

To assess the presence of fungal RNA in AMF-inoculated roots, reads not mapped to the tomato genome were additionally mapped against the *F. mosseae* reference transcriptome/genome. Reads from NMYC samples were also mapped against the same fungal reference as a control. Only a very small fraction of reads from NMYC samples mapped to *F. mosseae* (average < 0.1% of reads unmapped to the tomato genome), whereas MYC samples showed a consistently higher proportion of fungal-mapped reads, corresponding on average to approximately 1.6% of tomato-unmapped reads (Supplementary Table S19). This low value agrees with the low level of intensity of mycorrhization (M%, from 12.6% to 19.3%) and arbuscule abundance in whole root system (A% from 10.2% to 14.5%). However, the enrichment of fungal-mapped reads in MYC samples compared with NMYC controls supports the presence of fungal RNA associated with AM-colonized roots. Consistently, a limited but recurrent set of *F. mosseae* transcripts was detected across MYC samples (Supplementary Table S20), supporting fungal transcriptional activity in MYC root samples.

## Discussion

Tomato is considered a crop model for investigating AM symbiosis because it combines agronomic importance with extensive genetic and genomic resources, including mutant collections that enable functional analyses of host metabolic and signaling pathways (De Rose et al. 2025). Molecular analyses of tomato roots have shown broad transcriptional reprogramming during AM development, including regulation of genes encoding nutrient transporters, stress-responsive functions, hormone-related genes, and immunity-associated pathways (Zouari et al. 2014; Balestrini et al. 2019; Abdelsattar et al. 2024; also reviewed by Fiorilli et al. 2024). Beyond mRNA-level responses, RNAome-scale analyses in tomato roots have shown that AM symbiosis is associated with extensive regulation of alternative splicing and non-coding RNAs, supporting a multilayer symbiotic regulatory network (Zeng et al. 2023). More recent studies have further established tomato as a system for integrative analyses of AM symbiosis under stress conditions by combining transcriptomic and metabolomic approaches (Giovannini et al. 2025). In line with this, transcriptomic analyses of tomato roots under salinity showed AM-dependent regulation of pathways involved in stress perception and signaling, ion homeostasis, Ca^2+^ signaling, reactive oxygen species (ROS) detoxification, and SOS responses, supporting an important role of AM symbiosis in root stress adaptation (Marqués-Gálvez et al. 2025). All these studies highlight the importance of tomato as a crop model to investigate how host genetic and metabolic traits shape AM-related root responses.

In this context, our work focused on two tomato mutant lines with contrasting carotenoid-related phenotypes. While the mutation in *cyc-b7* has been well characterized, the genetic basis of the *7458-Y* phenotype remains unresolved. Among the candidate allelic variants identified in our study for *7458-Y*, two genes stand out for their potential involvement in carotenoid metabolism. *Solyc08g023460* has been identified as a putative target of *SlWRKY14*, a regulatory node of carotenoid and flavonoid biosynthesis in tomato (Li et al. 2025) and, thus, might be partially responsible for the carotenoid phenotype observed in *7458-Y* plants. According to the InterPro protein sequence analysis and classification database, the *Solyc08g023460* gene product belongs to the CDC50/LEM3 protein family, which may be involved in cell cycle control and lipid translocation in plants (Poulsen et al. 2008). Lipid biosynthesis and export are crucial to AM symbiosis, because AMF depend in part on host derived lipids (Gutjahr et al. 2012; Bravo et al. 2017; Jiang et al. 2017). Thus, perturbations in lipid-related pathways may affect the functionality of the plant-fungus interface and the maintenance of arbuscules. In this line, the *7458-Y* specific variant here identified could potentially affect membrane organization or vesicle-trafficking processes involved in arbuscule accommodation, although this remains to be experimentally tested. Additionally, *Solyc10g047130* is annotated as a glycine-rich RNA-binding protein-like *SlRZ1AL*. According to Li et al. (2022), *SlRZ1AL* participates in the regulation of carotenoid biosynthesis and metabolism, affecting tomato development and fruit ripening. However, the two allelic variants found in *7458-Y* are not located in the CDS region but in the 3’UTR. The direct implications of these and other variants on the carotenoid accumulation observed in *7458-Y* remain unstudied, and further, more focused experiments are required to elucidate this.

AM symbiosis has been shown to be able to induce carotenoid accumulation in plants, such as in lettuce leaves (Baslam et al. 2013), potato tubers (Tong et al. 2013), tomato fruits (Lidoy et al. 2025), as well as carotenoid metabolism in roots (Fester et al. 2002). Conversely, many apocarotenoids have previously been shown to influence AM establishment. Probably, the most relevant apocarotenoid might be SLs, since they are well-known to influence AM symbiosis (Lanfranco et al. 2018). Other important apocarotenoids are ABA and cyclohexenones and mycorradicins. For instance, Herrera-Medina et al. (2007) demonstrated that ABA contributes to promoting AM symbiosis establishment in tomato, playing an important role in the development of arbuscules. However, high exogenous ABA levels were shown to inhibit AM symbiosis, suggesting a dose-dependent regulatory effect (Charpentier et al. 2014). Moreover, Fester et al. (2002) found that *Zea mays* L. mutants deficient in carotenoid biosynthesis showed a significant reduction in mycorrhizal colonization and speculated on the importance of apocarotenoid accumulation in the establishment of AM symbiosis, with special attention to cyclohexenones and mycorradicins (Strack and Fester 2006). In agreement with this, apocarotenoid profiling in rice roots and shoots indicated that additional uncharacterized apocarotenoids are involved in AM symbiosis and that this metabolic pathway is extensively reprogrammed during the different stages of AM colonization (Votta et al. 2024). Overall, our data, together with previous literature, support the hypothesis that carotenoid accumulation in roots modulates mycorrhizal symbiosis, probably through its role as a precursor source for apocarotenoids. In this context, the contrasting carotenoid profiles of the mutant lines *cyc-b7* and *7458-Y* may reflect differences in carotene availability in roots, suggesting a possible link with the efficiency of AMF symbiosis observed in our experiments. The reduced β-carotene content in *cyc-b7* may reflect a lower availability of carotenoid-derived signaling molecules, whereas the higher β-carotene levels in *7458-Y* may be consistent with an increased biosynthetic flux towards these apocarotenoids, possibly in line with its higher arbuscule abundance. However, despite the contrasting AM colonization phenotypes of *7458-Y* and *cyc-b7* are consistent with their differences in root β-carotene content, these mutant lines may carry additional genetic changes. Therefore, we cannot exclude that part of the observed AM symbiosis and transcriptional phenotypes is influenced by carotenoid-independent background effects. The use of other carotenoid-accumulating mutants will be pivotal to establish a direct causal link between specific carotenoid alterations and AM symbiosis regulation.

In line with this, transcriptomic profiles of the two mutants, without AMF inoculation, differ from those of the Red Setter wild type. In particular, the strong transcriptomic reprogramming of *cyc-b7*, mainly in phytohormone and secondary metabolite pathways, suggests that, in roots, the mutation background may have affected not only the *SlCYC-B* gene but also related pathways. Concerning carotenoid-related pathways, in *cyc-b7*, the transcriptomic profile suggested that the upstream steps of the GGPP to be converted into lycopene (e.g., *SlPDS*, *SlZ-ISO*, *SlCRTISO*) are active, while the conversion of lycopene to carotene derivatives seems to be depleted.

Across the three considered genotypes, AMF inoculation triggered a genotype-dependent transcriptional response. Red Setter displayed a moderate number of DEGs in the MYC vs. NMYC comparison, *cyc-b7* showed a much stronger response (over 5000 DEGs), and *7458-Y* showed an intermediate response. Particularly, AMF inoculation was associated with the down-regulation of several genes encoding nutrient transporters in *cyc-b7*, together with reduced expression of *SlSYMRK* (a symbiosis receptor-like kinase). In *cyc-b7*, AMF inoculation elicited a response within the AM-responsive transporter set (two genes up-regulated vs. 22 down-regulated). This pattern might be consistent with a broad down-regulation of transport functions under MYC conditions and with the re-balancing between the direct and mycorrhizal nutrient-uptake pathways during AM symbiosis (Ferrol et al. 2019). The predominance of down-regulation of AM-responsive transporter genes in *cyc-b7* may may indicate a genotype-specific transcriptional response to AMF inoculation, rather than a classical activation of the mycorrhizal nutrient-exchange program (Balestrini et al. 2019; Banasiak et al. 2021).

In parallel, *cyc-b7* showed down-regulation of *SlSYMRK*, a key component of the common symbiosis signaling pathway required for AM development (Harrison 2005). Changes in *SYMRK* expression have been linked to altered AMF colonization in legumes (Gherbi et al. 2008), tobacco (Tan et al. 2013), maize (Zhou et al. 2025), and in tomato (Nair and Bhargava 2012). Thus, the lower colonization values observed in *cyc-b7* compared with *7458-Y*, together with a trend toward lower colonization relative to Red Setter (although not significant), suggest that *SlSYMRK* down-regulation should be interpreted as part of a genotype-dependent transcriptional response to AMF inoculation of this mutant.

By contrast, in *7458-Y*, AMF inoculation induced the expression of a gene encoding a tomato LysM receptor-like kinase (LYK12), a protein previously reported to influence AM colonization (Liao et al. 2018; Girardin et al. 2019). In addition, in *7458-Y*, together with the induction of *SlLYK12*, the gene *SlD27* was identified as significantly up-regulated in AM-inoculated roots. The *cis*/*cis*/*trans*-β-carotene isomerase D27 and the two carotenoid cleavage dioxygenases (CCD7 and CCD8) catalyze the early steps of SL biosynthesis (Kohlen et al. 2012; López-Ráez et al. 2015; Wang et al. 2024). Carlactone is then further oxidized to carlactonic acid (CLA) by the cytosolic cytochrome P450 enzymes, such as MORE AXILLARY GROWTH1 (MAX1) (Zhang et al. 2018; Wang et al. 2024). Thus, the expression of *SlD27* in the mutant roots in the presence of AMF suggests activation of the upstream step supplying the SL pathway, potentially increasing the availability of 9-*cis* β-carotene-derived precursors for downstream SL biosynthesis and, in turn, contributing to AMF recruitment and/or colonization. This outcome warrants further investigation, including the use of recently developed CRISPR/Cas deletion mutant panels targeting SL-related genes, including *SlD27* (Nicolia et al. 2025).

## Conclusions

Our results support a link between root carotenoid metabolism and the establishment of AM symbiosis in tomato. At the root level, the two mutant lines showed contrasting carotenoid profiles and distinct mycorrhizal colonization rates, suggesting that changes in carotene availability may affect the production of carotenoid-derived signals involved in AM establishment. In AMF-inoculated *cyc-b7* plants, mycorrhizal colonization was linked to a reduced β-carotene content, an extensive transcriptional reprogramming, and a down-regulation of key symbiosis- and transport-related genes. By contrast, AMF inoculated *7458-Y* plants showed higher β-carotene levels, greater arbuscule abundance, and AM-induced expression of genes related to AM establishment and SL biosynthesis, including *SlLYK12* and *SlD27*. These findings confirm that tomato carotenoid metabolism is closely connected with AMF colonization and highlight carotenoid-derived pathways as promising targets for future studies on the regulation of AM symbiosis. Further analyses aimed at functionally characterizing the 13 putative allelic variants underlying the *7458-Y* phenotype will be required to clarify the role of these polymorphisms in roots-AMF interactions in this tomato mutant.

## Supplementary Information

Supplementary Material 1.

Supplementary Material 2.

## Data Availability

The RNA-seq raw data are available in the NCBI Sequence Read Archive (SRA) at https://www.ncbi.nlm.nih.gov/sra and can be accessed with the BioProject accession number PRJNA1435166.
