# Supplementary material for: Arbuscular mycorrhizal symbiosis in tomato roots with a diverse range of carotene accumulation
Source: Mycorrhiza. 2026 Jun 10;36(3):32. doi: 10.1007/s00572-026-01277-0 (PMC13249782; doi:10.1007/s00572-026-01277-0)
Supplement: Supplementary file 2 — Supplementary Material 2. [file 572_2026_1277_MOESM2_ESM.pdf]

Arbuscular mycorrhizal symbiosis in tomato roots with a diverse range of carotene accumulation

Luca Giovannini[1]*^, José Eduardo Marqués-Gálvez[2,3]*, Fabiano Sillo[1], Domenico De Paola[2], Angelo Petrozza[4], Teresa Mango[4], Donato Melfi[4], Jian You Wang[5], Valentina Fiorilli[6], Filomena Carriero[4], Raffaella Balestrini[2]^

[1]National Research Council of Italy, Institute of Sustainable Plant Protection, Strada delle Cacce 73, 10135-Torino;

[2]National Research Council of Italy, Institute of Biosciences and Bioresources, Via Amendola 165/A, 70126-Bari;

[3]Mycology-Mycorrhizae-Plant Biotechnology Group, Department of Plant Biology, CEIR Campus Mare Nostrum (CMN), University of Murcia, Campus de Espinardo, 30100, Murcia, Spain;

[4] ALSIA Centro Ricerche Metapontum Agrobios, s.s. Jonica 106, km 448,2, Metaponto, MT 75010, Italy;

[5]Academia Sinica Biotechnology Center in Southern Taiwan, Agricultural Biotechnology Research Center, Academia Sinica, Tainan 71110, Taiwan;

[6]Department of Life Sciences and Systems Biology, University of Torino, Viale Mattioli 25, 10125, Turin, Italy.

*First authors

^Corresponding authors

luca.giovannini@cnr.it

raffaellamaria.balestrini@cnr.it

Supplementary Figures

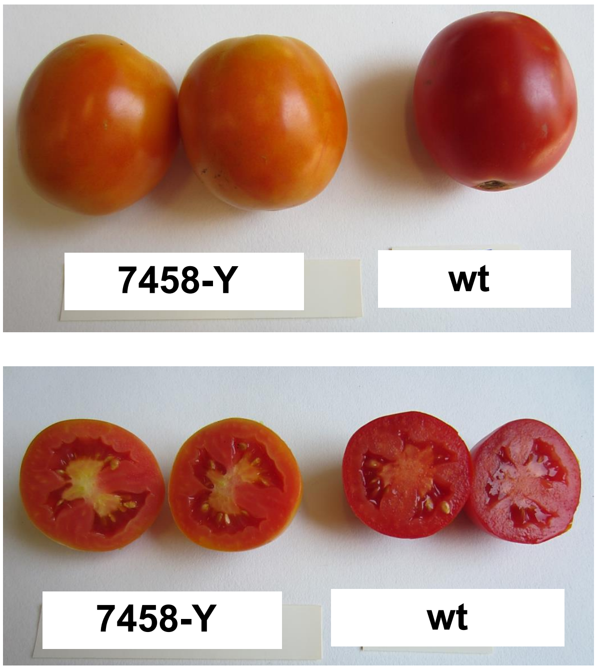

Supplementary Fig. S1 Fruit phenotype of the tomato mutant *7458-Y* compared with the wild-type Red Setter. Representative images of mature fruits (upper panel) and transverse fruit sections (lower panel) showing the altered fruit pigmentation of *7458-Y* relative to Red Setter (wt)

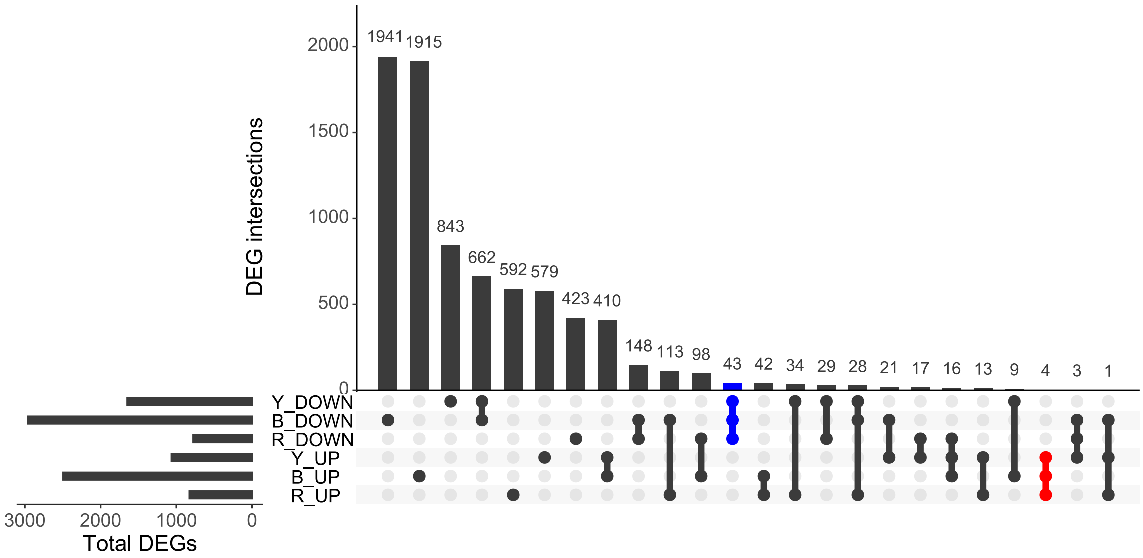

Supplementary Fig. S2 UpSet plot of DEGs identified in AMF-inoculated tomato roots. UpSet plot showing overlaps among differentially expressed genes (DEGs) identified in AMF-inoculated plants (MYC) compared with their corresponding not-inoculated controls (NMYC) within each tomato genotype: Red Setter (R), *cyc-b7* (B), and *7458-Y* (Y). Up- and down-regulated DEGs are shown separately for each genotype. Horizontal bars indicate the total number of DEGs in each set, while vertical bars indicate the size of each intersection. The highlighted intersections indicate DEGs commonly down-regulated (blue) or up-regulated (red) across the three MYC vs. NMYC comparisons
